# Dr. Nelson W. Williams

**Published:** 1901-08

**Authors:** 


### DR. NELSON W. WILLIAMS.

DR. NELSON W. WILLIAMS was born at West Liberty, Logan County, Ohio, October 23, 1834, and died at Nice, France, May 5, 1901.

Dr. Nelson commenced the study of dentistry with Dr. Harris, of West Liberty, Ohio, about 1855. During the succeeding five years he diligently prepared himself to enter the Ohio College of Dental Surgery, which he did in 1860. After taking one course he started practice at West Liberty, dividing his time between that place and Kenton, Ohio. After two years of practice he returned to college, when he graduated in 1863. Shortly after this he entered into partnership with Drs. Jonathan Taft and George Watt, of Xenia, Ohio.

In 1871 he was invited to enter the practice of Dr. Slayton, Sr., of Florence, Italy. This he accepted, going there in 1872, but after a year's stay in Florence he decided to go to Geneva, Switzerland, where he bought out the practice of Dr. George W.

Field, now of London. After several years of hard work his health failed, and he had to go to the Riviera to recuperate. Finding he could not endure the rigors of the Swiss winters, he decided to dispose of his Geneva practice and locate at Nice, where he practised until his death.

He was a member of the Ohio State Dental Society and of the Mad River Dental Society, from both of which he received a specially gratifying testimonial upon his leaving Ohio for Europe. While at Geneva he was one of the five founders of the American Dental Society of Europe, and while failing health of late years had prevented his regular attendance at its meetings, he always had a warm place in his heart for its welfare and success, as was testified by his cheery letter read at its last meeting at Cologne. As a matter of history it may be interesting to note that Drs. Watt and Williams were the first to make crystal gold in the United States, and Dr. Williams was the one to introduce it to the profession in Europe, and he also showed his lamented friend, Dr. de Trey, of Vevey, how to produce it, the outcome of which is the solila gold of to-day.

Dr. Williams was the pioneeer of American dentistry on the Riviera, and those who had a close acquaintance with him will sadly miss his kindly genial greeting, and will join with the wife and daughters who now mourn the loss of husband and father in bowing to the stern fate that deprives them of a dear good friend and our profession of one of its pioneers and most skilful and conscientious representatives.

He was laid to rest in the French Cemetery at Cancade, near Nice, in the presence of a great concourse of sorrowing friends and all the members of the French Dental Society of the Riviera. *Requiescat in pace.*

